# Perspective on *in vivo* SPME for human applications: Starting from monitoring doxorubicin during lung chemo-perfusion

**DOI:** 10.1016/j.jpha.2023.12.008

**Published:** 2023-12-12

**Authors:** Wei Zhou, Runshan Will Jiang, Barbara Bojko, Janusz Pawliszyn

**Affiliations:** Department of Chemistry, University of Waterloo, Waterloo, ON N2L 3G1, Canada; Department of Pharmacodynamics and Molecular Pharmacology, Collegium Medicum in Bydgoszcz, Nicolaus Copernicus University in Torun, 85-089, Bydgoszcz, Poland; Department of Chemistry, University of Waterloo, Waterloo, ON N2L 3G1, Canada

## Abstract

• *In vivo* SPME is a powerful technique for obtaining comprehensive information.
• Non-destructive and mini-invasive sampling technique with quantitative capability.
• Application for *in vivo* monitoring drug concentration in human tissue.
• Noninvasive applications on *in vivo* human breath and skin analysis.
• Future perspective on SPME devices, coupling methods and applications.

## Graphical abstract

**Figure:**
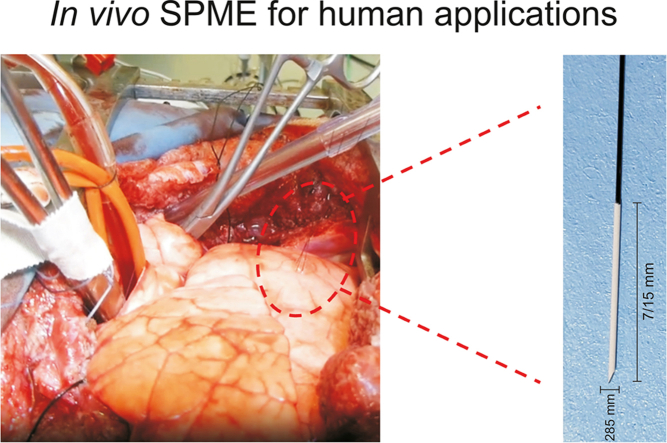

## 1 Introduction

*In vivo* analysis provides more accurate information for indicating or predicting the processes occurring in the complex living organisms compared with the *ex vivo* study. Combining *in vivo* sampling with liquid chromatography (LC)/gas chromatography-mass spectrometry (GC-MS) analysis is the most powerful technique for obtaining comprehensive information, as it can yield both targeted and nontargeted data with quantitative capability. Microdialysis (MD) is a widely used method for *in vivo* sampling over the years, especially valued for its ability to collect samples continuously over time. However, MD analysis is limited by a few drawbacks that create the need for complementary and/or alternative sampling methods. First, sampling of nonpolar compounds can present a problem in MD analysis. As nonpolar compounds are typically highly bound to tissue matrix, they are only available in very low free concentration levels that limit their detection and quantitation. In some cases, such compounds can also adsorb onto the membrane and/or tubes used in MD. Secondly, collected samples require subsequent sample preparation, such as liquid extraction or solid-phase extraction, prior to chromatographic separation and MS analysis. In addition to being time-consuming and labour intensive, these additional sample preparation steps can also lead to the decomposition of some unstable compounds, and thus negatively impact the accuracy and precision of the final data.

Solid-phase microextraction (SPME), in which a small amount of extraction phase is immobilized onto a solid support such as a fiber to extract analytes from samples, combines sampling, sample preparation, and enrichment into a single step. In human applications, SPME was first successfully applied for *in vivo* analysis of human breath, saliva, blood in vein and skin surfaces. The subsequent development of biocompatible coating materials using biosafe coating and supporting material has expanded the use of SPME and enabled *in vivo* analysis of tissue through direct insertion of fibers into the tissues. The miniaturized nature of SPME enables minimally invasive sampling, while its non-exhaustive extraction mechanism causes minimal perturbation to the living system, as only a small portion of analytes is extracted via free concentration [1]. These advantages allow researchers to acquire ‘snapshots’ of unique biological processes taking place in regions not otherwise easily sampled via traditional methods. For instance, they have permitted an in-depth metabolomics study with high temporal and spatial resolution by *in vivo* SPME sampling of brain tissue of awake and freely moving rats [2]. The using of *in vivo* SPME instead of regular tissue biopsy can also alleviate animal suffering and reduce the consumption of animal life.

**Fig. 1:**
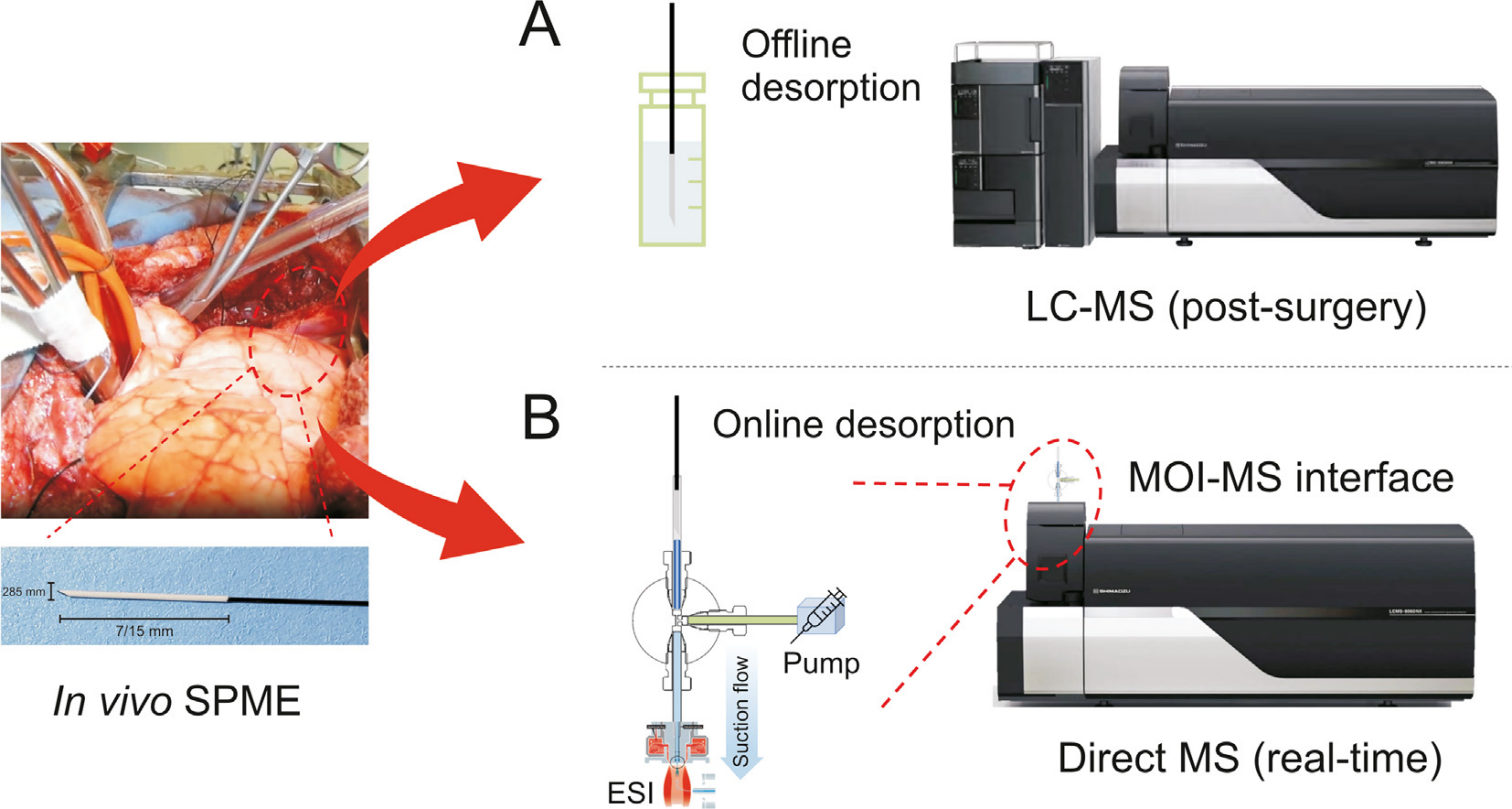
Coupling *in vivo* solid-phase microextraction (SPME) with (A) liquid chromatography-mass spectrometry (LC-MS) for post-surgery analysis or (B) direct MS for real-time analysis during surgery. ESI: electrospray ionization; MOI: microfluidic open interface.

After more than 10 years adventures, going through *ex vivo* biofluidic, tissue, *in vivo* mouse, rat, dog and monkey studies, *in vivo* SPME has been finally used for human applications in the monitoring of doxorubicin (DOX) concentration during the *in vivo* lung perfusion (IVLP) surgery (Fig. 1A) [3]. Pulmonary metastases are secondary cancerous cells that develop in approximately 30% of patients with malignant tumors. IVLP is used to locally deliver DOX, a powerful anti-cancer drug that can cause multi-organ toxicities, to isolated lung tissue during surgical resection in an effort to reduce its toxicity to other organs while enabling the use of higher doses in the targeted organs. However, during IVLP, the concentration levels and distribution of DOX in different parts of the lung must be precisely controlled so as to prevent insufficient application or excessive dosing. While traditional methods of monitoring that rely on tissue biopsy collected from the terminal parts of the organ are invasive and cannot provide spatial resolution, the successful implementation of *in vivo* SPME-LC-MS for analysis of lung tissue of two patients undergoing IVLP surgery for a period of 3 h has enabled quantitative results with both temporal and spatial resolution. This ground-breaking achievement thus opens new horizons for *in vivo* SPME bioanalytical applications in medicine and beyond. Within this context, we provide here a brief future perspective on *in vivo* SPME focusing on human applications.

## 2 Perspectives on the future of *in vivo* SPME devices

In the aforementioned IVLP application, a commercially available biocompatible SPME fiber (diameter, 285 μm; coating thickness, 45 μm; coating length, 15 mm) using a mixed-mode coating (C8 + benzenesulfonic acid particles embedded in polyacrylonitrile) while affixed to a nitinol support was used. The device can be further optimized to maximize its applicability for *in vivo* tissue analysis.

Firstly, although the nitinol support is chemically and physically stable, it can be easily bent during insertion into lung tissue, thus requiring special care during the operation. Additionally, traversing into resistant tissue such as muscle requires the use of a sheath needle or guide cannula, which adds an inconvenient step to the workflow. Given that nitinol is a metal characterized by a darker hue and the device is small, doctors observed that particular care needs to be taken to not lose fibers placed in the tissue, especially when multiple fibers are used simultaneously. Further optimization of the device should include both the use of a stronger support material such as a medical-grade stainless-steel needle, and the addition of a suitable holder on top of the devices to facilitate visual tracking and removal. Secondly, the mixed-mode coating used for this application is usually activated by organic solvents prior to sampling to ensure better extraction efficiency and reproducibility. However, sterilization of the fibers prior to insertion by autoclave deactivates the coating by removing the solvents, thus resulting in lower efficiency and reproducibility. In this regard, future directions should take into consideration both the development of new fiber sterilization methods and the development of new coating materials that do not require activation via solvents. Finally, the continued development of highly efficient coating materials for SPME is important for the analysis of different target analytes. Nowadays, most of the coating materials for SPME are primarily suitable for extraction of nonpolar compounds; the development of biocompatible coating materials with good extraction efficiency towards polar analytes would greatly increase the applicability of SPME.

## 3 Direct coupling of *in vivo* SPME with MS: enabling real time monitoring during surgery

In the aforementioned application, samples extracted via *in vivo* SPME were submitted to LC-MS analysis after surgery for investigating the general trends of metabolic processes. However, for point-of-care analysis, particularly for individuals with advanced-stage cancer, metabolic processes can vary significantly among patients due to the disparities in their physiological conditions. In this sense, real-time monitoring of anti-cancer drugs during surgery could provide useful information to the medical team, enabling more precise adjustments to dosage in different targeted areas throughout the surgery while also helping inform doctors on other decisions related to the surgical procedure. For example, IVLP requires re-perfusion with clean blood following DOX delivery to remove any drug residue in tissue after chemotherapy; knowing the exact drug concentration left in the tissue would aid physicians in timing the re-perfusion step adequately.

While ambient MS techniques developed for real-time MS analysis, such as the intelligent knife and the mass spectrometer pen, are available for diverse medical applications, these are limited to providing qualitative information on cut tissue (knife) or the surface of tissue (pen). Coupling *in vivo* SPME to direct/ambient MS analysis could provide quantitative data with spatial resolution via insertion of SPME needles into the targeted tissue areas followed by subsequent instrumental analysis. Taking advantage of the sample clean up function of SPME, rinsing fibers with water for a few seconds following extraction to remove nonspecific attachments on the coating would be sufficient for direct desorption and analysis by MS. For such applications, the newly developed automated microfluidic open interface (MOI) could be employed to facilitate the instrumental workflow following extraction from tissue (Fig. 1B) [4]. Using this automated interface, a medical professional would just need to place the rinsed fiber into an open chamber and then remove it after 10 s; data would load onto the computer software interface in real time to quickly aid the team in adjusting concentration levels as needed. In addition to the MOI, other direct/ambient MS techniques such as nano electrospray ionization (ESI)-MS or probe ESI (PESI)-MS can also be considered.

The biggest challenge posed by the proposed direct MS technique is that the MS instrument should be placed in close proximity to where the extractions are being performed, either in the surgery room or somewhere adjacent. Traditional MS instruments can be quite large and noisy due to the vacuum pumps; as such, the use of portable MS instruments might be a solution to this limitation.

## 4 Future applications: from targeted drug monitoring to untargeted metabolomics studies

For *in vivo* applications, SPME has its unique feature when compared with other techniques. At present, only two distinct applications of *in vivo* SPME have been validated for human use, DOX monitoring during IVLP and, more recently, untargeted profiling of human brain tissue [5]. The development and implementation of *in vivo* SPME for human applications is not a venture initiated from ground zero; SPME has already been employed in a variety of applications using large animal models for different purposes such as monitoring of folinic acid, 5-fluorouracil, and oxaliplatin during IVLP, quality assessments of graft (such as kidney and liver) transplantations, and monitoring of dynamic changes during *ex vivo* heart perfusion. Recent human applications provide robust evidence that translations of *in vivo* SPME applications using large animal models (such as pig and monkey) to human studies are feasible and bolster our confidence in expanding the use of this technology for various future applications.

In addition to *in vivo* SPME sampling for various applications, integration with advanced analytical technologies, like direct MS for rapid quantification of target analytes, or LC/GC coupled with high-resolution MS and ion mobility-MS for untargeted metabolomics screening, can yield comprehensive insights into living systems.

## CRediT author statement

**Wei Zhou**: Writing - Original draft preparation, and Reviewing and Editing; **Runshan W. Jiang**: Writing - Reviewing and Rditing; **Barbara Bojko**: Writing - Reviewing and Editing; **Janusz Pawliszyn**: Supervision, Writing - Reviewing and Editing.

## Declaration of competing interest

The authors declare that there are no conflicts of interest.
